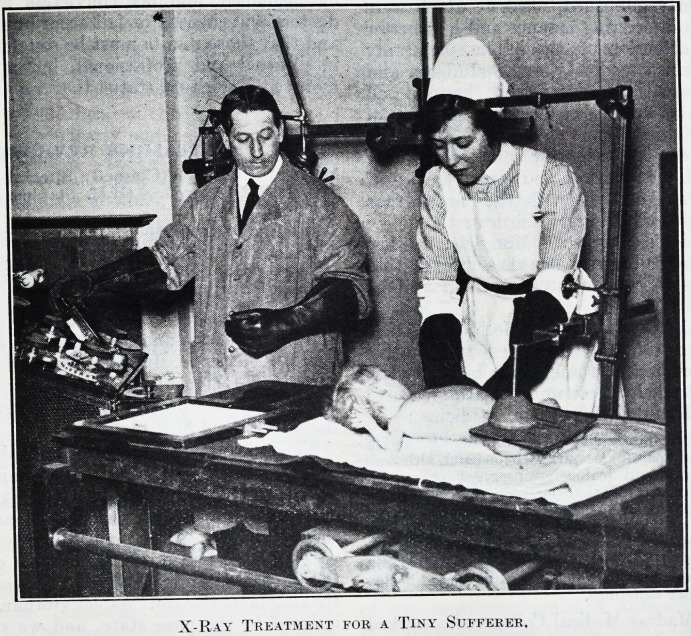# The Battle for Infant Life

**Published:** 1924-05

**Authors:** 


### THE BATTLE FOR INFANT LIFE.

To assist the Infants' Hospital, Vincent Square, in its battle for infant life, Princess Mary, its President, paid a visit on April 14 to inaugurate the pound scheme, which is an appeal to the nation for money for the general extension of the hospital, greater facilities for research, and many other improvements of which this excellent and progressive institution stands in need. The meeting was held in a marquee in Alfred Street, gay with flowers and crowded with enthusiastic supporters. Specimens of novel collecting receptacles were exhibited—a hat resembling the "Mad Hatter's," and a specially designed box with a figure of a nurse and baby. There is also a special medal for box and card helpers collecting a guinea and upwards. All the energy and originality put into this campaign deserves success, and the Chairman, Mr. Gomer Berry, was supported by a distinguished company, including the Minister of Health, Miss Ishbel MacDonald, Lady Mond and Dr. Eric Pritchard, Medical Director, whose statement that strides in medical knowledge had practically doubled the expenses of the hospital showed what a problem had to be faced.

X-Ray Treatment for a Tiny Sufferer.

### Educating the Mother.

Mr. Berry spoke of the deep interest and unfailing sympathy Princess Mary had displayed as President, her receptiveness to any suggestion the Committee had put forward, and her more than willingness, in spite of many pressing calls on her time, to help. The growth of the work was, he said, remarkable. In 1903, the year of its foundation for the treatment of young babies suffering from the diseases and disorders of nutrition, only 138 patients were admitted and the expenditure was £837 ; in 1922 there were 244 in-patients and 3,468 out-patient attendances. Last year the latter were doubled and the total expenditure was over £10,000. Four children had to be sent away for every one taken. The Minister of Health said that the child welfare work undertaken by thousands of voluntary workers was watched with sympathy by the Ministry, and they were prepared to give it every encouragement. He was particularly interested in the preventive side of the work and in its educational aspect. It should be its aim to build up a motherhood capable of looking after its children and in a position to give them the essential nourishment of life. He welcomed every effort to educate the mothers of the country, who should be intellectually and economically the safest and kindest guardian of their children. Miss Ishbel MacDonald, who made her first speech on a public platform in London, pointed out that it was particularly the children who escaped death but were faced with suffering and perhaps permanent ill-health for whom we had to provide. The research work done at the Infants' Hospital affected not only Westminster; it affected the whole country. Moreover, the women trained at this hospital went out and by their knowledge helped the babies and mothers throughout the land. Her Royal Highness then received purses amounting to over £280, and was shown a list of subscribers and helpers with a total of £2,826.

### MODELLING BY PHOTOGRAPHY.

The recent invention of a machine which, through the medium of special photographs, is able exactly to model the contours of the object may undoubtedly have a most revolutionary effect upon portrait sculpture. But in the world of science, too, its possible application is wide. There are many objects, particularly those of interest to medicine and biology, which are so delicate and the natural haracters of which last so short a time that modelling and cast-making in the usual sense are extremely difficult, if not impossible. Since obtaining the "cameograph," as the new invention is named, involves no more stress to the subject than the taking of an ordinary photograph, the new opportunity of recording accurately dimensional details is obvious. Various medical museums are justly proud of their beautiful wax and plaster models of pathological rarities. Photographic modelling may well allow a revival of this very excellent method of anatomical and allied teaching.

## Figures and Tables

**Figure f1:**